# Satisfaction Levels in Doctors About Workplace Environment During the COVID-19 Pandemic: Experiences from Tertiary Hospitals in Dhaka, Bangladesh

**DOI:** 10.7759/cureus.43897

**Published:** 2023-08-22

**Authors:** Sifat Sharmin, Fahmida Khanom, Zerin S Rahman, Tanvir M Tonik, Golam Abbas

**Affiliations:** 1 Public Health and Hospital Administration, National Institute of Preventive and Social Medicine (NIPSOM), Dhaka, BGD; 2 Virology, National Institute of Preventive and Social Medicine (NIPSOM), Dhaka, BGD; 3 Maternal and Child Health, National Institute of Preventive and Social Medicine (NIPSOM), Dhaka, BGD; 4 Oncology, United Hospital Limited, Dhaka, BGD; 5 Occupational and Environmental Health, National Institute of Preventive and Social Medicine (NIPSOM), Dhaka, BGD

**Keywords:** work environment, msq scale, job satisfaction, doctors, covid-19

## Abstract

Background: The satisfaction level of doctors regarding the workplace environment signifies both the psychological and physical environment. One of the many challenges to conquer was the adaptation to a steadily changing working environment and the development of a proper working environment during the COVID-19 pandemic.

Objectives: The objective of this study was to evaluate the level of satisfaction of doctors regarding the workplace environment.

Methods: A cross-sectional study was conducted from January to December 2020. A total of 217 conveniently selected doctors working at selected tertiary hospitals in Dhaka city were interviewed using a pretested, structured questionnaire. The Minnesota Satisfaction Questionnaire (MSQ) was used to assess the level of job satisfaction on a 5-point Likert scale consisting of 20 items. The percentile score was used to categorize the respondents as highly satisfied (75 and above), averagely satisfied (26 to 74), and dissatisfied (below 25). Bivariate and multivariate analyses were performed.

Result: Among the 217 respondents, the total mean MSQ score was 3.62±0.23 regarding job satisfaction. About two-thirds of the respondents (63.1%) reported an average level of satisfaction. More than two-thirds of respondents (69.6%) expressed high satisfaction regarding the physical work environment, while the majority of respondents (93.1%) expressed high satisfaction with the psychosocial work environment. However, no significant association was found between outcome and input variables (p>0.05).

Conclusion: The study findings showed that satisfaction regarding the psychological environment was higher among the respondents than that of physical working conditions. Evidence-based measures are to be addressed in hospitals to achieve the optimum level of satisfaction among doctors during pandemics.

## Introduction

A poor work environment in hospitals may hamper work performance and promote pessimistic attitudes towards patients and colleagues. Job satisfaction has proven to be an important factor influencing productivity and is of great interest to healthcare organizations [1]. To survive in the medical market, it is important to provide patients with quality service. This is successfully achieved when a country can ensure the satisfaction of healthcare workers [2]. During the COVID-19 pandemic, there was a tremendous increase in workload, a strict protocol for maintaining social distancing that led to a high level of dissatisfaction, a lack of availability of PPE and adequate equipment, and a lack of a sense of fulfillment, all of which impacted job satisfaction. In the health market, the satisfaction of doctors influences their performance. Job satisfaction can be measured by a favorable working environment, workload, payment, and job security [3]. 

The COVID-19 pandemic has significantly influenced the health system worldwide. The working life of doctors continues to change significantly and will change further in the next few years [4]. In the health environment, doctors’ satisfaction is directly related to quality of service and patient satisfaction. Patients are primarily affected by their interactions with doctors. If doctors are not satisfied, the results can be tragic [5]. Job satisfaction is also important to the future recruitment of new doctors and retention of existing doctors, in addition to the productivity and quality of the services provided by the doctors, who are an essential and integral component of our medical care system [6]. To meet the standard quality of care, doctors need a working environment that allows them to work freely without problems that may restrain them from performing up to their full potential [7]. Doctors can provide high-quality healthcare services to patients when they are respected internally and satisfied with their work environment [8]. This study aimed to assess how satisfied doctors were with their work environment to deliver quality services during the COVID-19 pandemic.

## Materials and methods

A cross-sectional study was conducted from January 1 to December 31, 2020, to assess the satisfaction level of doctors regarding their work environment in select tertiary hospitals (Dhaka Medical College and Hospital (DMC), Sir Salimullah Medical College and Hospital (SSMC), and Shaheed Suhrawardy Medical College and Hospital (ShMCH)) during the COVID-19 pandemic. A non-probability convenient sampling technique was used to collect data from 217 respondents (doctors of selected tertiary hospitals). The sample size was calculated using the n = z2pq/d2 formula. The data collection was carried out using a pretested structured questionnaire through face-to-face interviews after obtaining informed written consent from each participant.

The questionnaire was composed to collect data regarding the physical environment, psychological environment, personal satisfaction, and job security, along with the socio-demographic information of the respondents. The Minnesota Satisfaction Questionnaire (MSQ) was used to assess the level of job satisfaction. The questionnaire included 20 items. Each item refers to reinforcement in the work environment. Five response alternatives were presented for each item: very dissatisfied; dissatisfied; neither (dissatisfied nor satisfied); satisfied; and very satisfied. Previous research yielded excellent coefficient alpha values (ranging from 0.85 to 0.91) that have made the MSQ scale a well-known and stable instrument over time. The percentile score was used to categorize the respondents as highly satisfied (75 and above), averagely satisfied (26 to 74), and dissatisfied (below 25). 

After completion of data collection, to maintain consistency, the data were checked and edited manually and verified for any omission, error, or irrelevance before tabulation. Data were coded, entered, and analyzed on a laptop using SPSS Statistics version 23 (IBM Corp., Armonk, NY, USA). Bivariate and multivariate analyses were performed to identify any association between outcome and input variables. The findings of the study are presented by frequency percentage in tables and graphs. The mean and standard deviations for continuous variables and frequency distributions for categorical variables were used to describe the characteristics of the total sample. The calculated sample size was 198. To minimize dropout, we made a 10% increase, and the sample size was 217. The privacy and confidentiality of each participant were strictly maintained. Ethical approval was obtained from the Institutional Review Board (IRB) of the National Institute of Preventive and Social Medicine (NIPSOM), Dhaka, Bangladesh (approval no. NIPSOM/IRB/2020/1225).

## Results

This study's findings showed that half of the respondents (50%) were from the age group 31 to 35 years, where the mean (± SD) age was 33.2 (± 4.2). About three-fifths of the respondents (58.1%) were male. Most of the respondents were Muslims (85.3%) and married (84.3%). The participants had different educational qualifications; among them, 17.5% had only an MBBS degree, while others had additional degrees (Table 1).

**Table 1:** Socio-demographic profile of the respondents (n=217)

Socio-demographic profile	Frequency (n)	Percentage
Age (years)		
≤30	59	27.2
31-35 and 36-40 >40 Mean± SD Min-max	108 38 12 33.2± 4.2 28-50	49.8 17.5 5.5
Gender		
Male/Female	126/91	58.1/41.9
Religion		
Muslim/Hindu	185/32	85.3/14.7
Marital status		
Married/Unmarried	183/34	84.3/15.7
Educational Qualification		
MBBS/FCPS/MS/MD/Others	38/32/5/14/128	17.5/14.7/2.3/6.5/59.0

**Figure 1:**
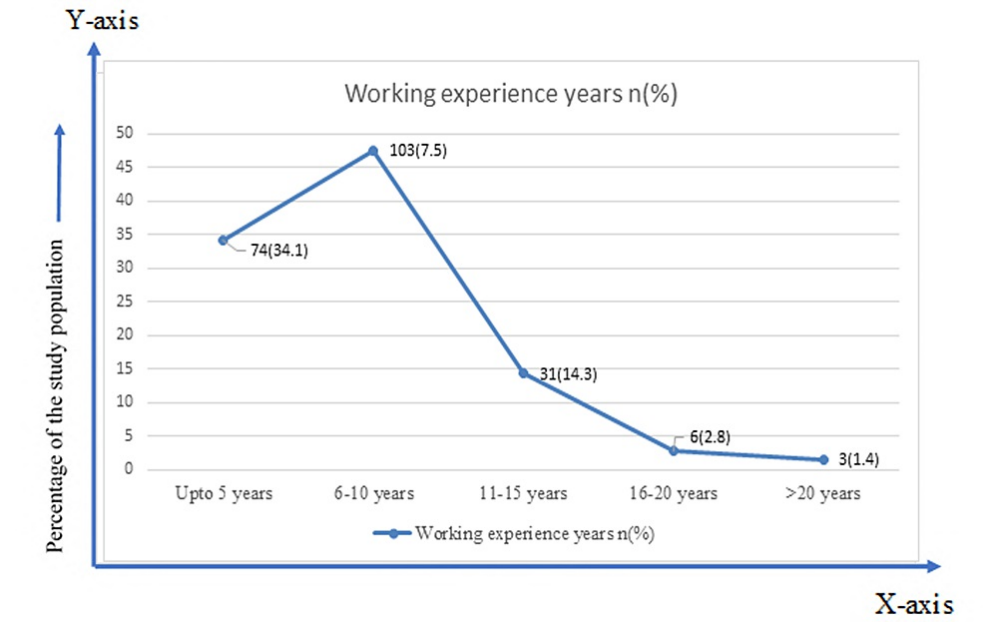
Working experience of the respondents in years (n=217)

About half the respondents (47.5%) had a working experience of six to 10 years (Figure 1).

Our findings showed that mean MSQ scores were almost similar in different work experience groups. The respondents with the highest mean (±SD), which was 3.68 (± 0.14) had a work experience of 16 to 20 years. To detect the statistical significance, an ANOVA test was done. The differences were statistically non-significant (p>0.05) (Table 2).

**Table 2:** Mean MSQ score regarding job satisfaction in different work experience groups (n=217) MSQ: Minnesota Satisfaction Questionnaire

Experience	Mean ± SD	p-value
Up to 5 years	3.63 ± 0.23	0.322
6-10 years	3.59 ± 0.24	
11-15 years	3.67 ± 0.23	
16-20 years	3.68 ± 0.14	
>20 years	3.47 ± 0.20	

According to the study findings, the highest mean (± SD) MSQ score was 4.68 (± 0.62), obtained for activity by the respondents. The lowest mean (± SD) was 2.19 (±1) for working conditions. The general mean MSQ score was (± SD) 3.62 (± 0.23) (Table 3).

**Table 3:** Doctors satisfaction analysis for each question on the MSQ (n-217) MSQ: Minnesota Satisfaction Questionnaire

Item	Mean ± SD
Ability utilization	3.32 ± 1.14
Achievement	2.64 ± 1.09
Activity	4.68 ± 0.62
Advancement	3.32 ± 1.17
Authority	4.53 ± 0.82
Company policies and practices	3.19 ± 1.16
Compensation	2.65 ± 1.06
Co-workers	3.13 ± 1.13
Creativity	4.34 ± 0.85
Independence	4.01 ± 0.98
Moral values	4.18 ± 0.98
Recognition	3.78 ± 0.96
Responsibility	3.53 ± 1.08
Job security	3.35 ± 0.99
Social service	4.16 ± 0.94
Social status	4.52 ± 0.88
Supervision-human relation	3.10 ± 1.13
Supervision-technical	3.54 ± 1.12
Variety of work	4.22 ± 0.90
Working condition	2.19 ± 1.00
General	3.62 ± 0.23

**Figure 2:**
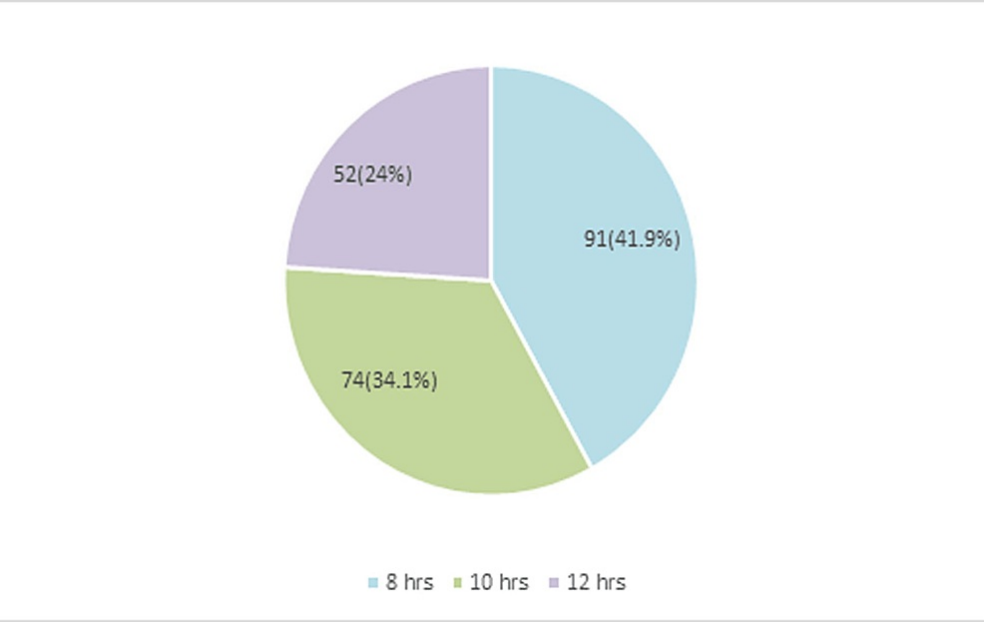
Working hours of the respondents (n=217)

Around two-fifths (41.9%) of the respondents worked for eight hours (Figure 2).

The study's findings were compared to the mean MSQ scores in the three different hospitals. The highest mean (±SD) was 3.65 (±0.21), found in SSMC (Table 4).

**Table 4:** Mean MSQ score in 3 different hospitals (n-217) MSQ: Minnesota Satisfaction Questionnaire, DMC: Dhaka Medical College and Hospital, SSMC: Sir Salimullah Medical College and Hospital, ScMCH: Shaheed Suhrawardy Medical College and Hospital

Name of hospital	Mean ± SD
DMC (n=67)	3.61 ± 0.26
SSMC (n=70)	3.65 ± 0.21
ShMC (n=80)	3.60 ± 0.23

About two-thirds of the respondents (63.1%) reported an average level of satisfaction. More than two-thirds of respondents (69.6%) expressed high satisfaction regarding the physical work environment, while the majority of respondents (93.1%) expressed high satisfaction with the psychosocial work environment (Table 5).

**Table 5:** Level of satisfaction (n=217)

Type of Satisfaction	High	Average
General satisfaction	36.9% (n=80)	63.1% (n=137)
Physical environment	30.4% (n=66)	69.6% (n=151)
Psychosocial environment	93.1% (n=202)	6.9% (n=15)

**Figure 3:**
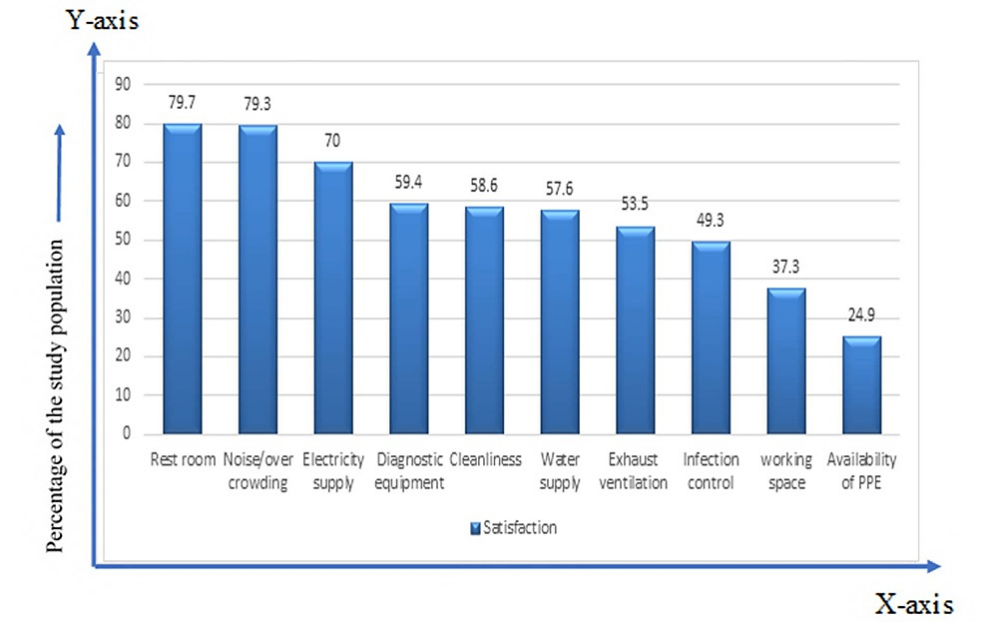
Satisfaction regarding workplace facility (n=217)

Around 79.7%, 79.3%, 59.4%, and 58.6% of the respondents were reported to be satisfied regarding the rest room, noisy/overcrowded working place, diagnostic equipment, and cleanliness, respectively. Satisfaction regarding the availability of PPE was 24.9% (Figure 3).

**Figure 4:**
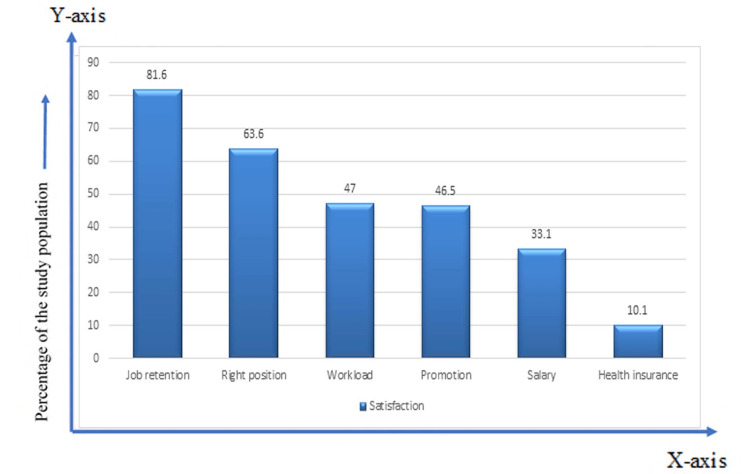
Personal satisfaction of the respondents (n=217)

The majority of respondents (81.6%) were satisfied with job retention. One-third of the respondents (33.1%) were satisfied with their salary, and 10.1% of the respondents were satisfied with the health insurance facility (Figure 4).

In response to a question regarding security services for doctors in hospitals, 34 (15.66%) MBBS doctors, 27 (12.44%) FCPS doctors, 13 (5.99%) MDs, and four (1.84%) MS doctors reported that they had security services in their workplace. Whereas four (1.84%) MBBS doctors, five (2.30%) FCPS doctors, one (0.46%) MD, and one (0.46%) MS doctor worked in an environment without security services (Table 6).

**Table 6:** Association between educational status and security services for doctors

Educational status	Security service for doctors			p-value
	Yes	No	Total	0.902
MBBS	34 (15.66%)	4 (1.84%)	38 (17.51%)	
FCPS	27 (12.44%)	5 (2.30%)	32 (14.75)	
MS	4 (1.84%)	1 (0.46%)	5 (2.30%)	
MD	13 (5.99%)	1 (0.46%)	14 (6.45%)	
Others	112 (51.61%)	16 (7.37%)	128 (58.99%)	
Total	190 (87.56)	27 (12.44%)	217 (100%)	

The study revealed that eight (3.69%) doctors with ages ≤30 had health insurance, and 14 (6.45%) doctors with ages 31 to 35 had health insurance. On the other hand, 48 (22.12%) doctors aged ≤30, 76 (35.02%) doctors with ages between 31 and 35, 30 (13.82%) doctors with ages between 36 and 40, and 10 (4.61%) aged >40 years had no health insurance policy (Table 7).

**Table 7:** Association between age and health insurance for doctors

Age	Health insurance for doctors				p-value
	Yes	No	No comment	Total	0.005*
≤30	8 (3.69%)	48 (22.12%)	3 (1.38%)	59 (27.19%)	
31 - 35	14 (6.45%)	76 (35.02%)	18 (8.29)	108 (49.77)	
36 - 40	-	30 (13.82%)	8 (3.67%)	38 (17.51%)	
>40	-	10 (4.61%)	2 (0.92%)	12 (5.53%)	
Total	22 (10.1%)	164 (75.58%)	31 (14.29%)	217 (100%)	

The mean MSQ score regarding job satisfaction is almost similar in different age groups. Among the respondents, the highest mean ± SD score is found (3.66 ± 0.23) within the age group 36 to 40, and the lowest score is (3.61 ± 0.24) within the age group up to 30, and similarly in the 31 to 35 age group and respondents above 40 years of age (3.65 ± 0.19). The total mean MSQ score regarding job satisfaction in different age groups is 3.62 ± 0.23. To see the impact of different age groups on job satisfaction, a one-way ANOVA test was done. The differences were statistically non-significant (p=0.686) (Table 8).

**Table 8:** Mean MSQ score regarding job satisfaction in different age groups (n=217) MSQ: Minnesota Satisfaction Questionnaire

Age (in years)	Mean ± SD	p-value
≤30	3.61 ± 0.24	0.686
31-35	3.61 ± 0.24	
36-40	3.66 ± 0.23	
>40	3.65 ± 0.19	
Total	3.62 ± 0.23	

The mean MSQ score regarding job satisfaction in males and females is almost similar. In the male group, the mean ± SD is 3.61 ± 0.24, and in the female group, the mean is ± SD (3.62 ± 0.23). To see the impact of gender variation on job satisfaction, an unpaired t-test was done, and the result was found to be non-significant (p=0.860) (Table 9).

**Table 9:** Mean MSQ score regarding job satisfaction in males and females (n=217) MSQ: Minnesota Satisfaction Questionnaire

Gender	Mean ± SD	p-value
Male	3.61 ± 0.24	0.860
Female	3.62 ± 0.23	

The mean MSQ score in the different marital statuses regarding job satisfaction is almost similar: the mean ± SD in married and unmarried groups is 3.62 ± 0.24 and 3.61 ± 0.21, respectively. The differences are statistically non-significant. To find out the statistical significance, an unpaired t-test was done (p=0.852, non-significant as p>0.050 (Table 10).

**Table 10:** Mean MSQ score in different marital statuses regarding job satisfaction (n=217) MSQ: Minnesota Satisfaction Questionnaire

Marital status	Mean ± SD	p-value
Married	3.62 ± 0.24	0.852
Unmarried	3.61 ± 0.21	

The mean MSQ score at different education levels regarding job satisfaction is almost similar. Among the respondents, the highest mean ± SD score (3.68±0.22) is found in only MBBS respondents. For participants who have additional FCPS, MS, and MD degrees, the scores were (3.66±0.24), (3.53 ± 0.23) and (3.68 ± 0.17), respectively. In other educational qualifications (M.Phil., diploma, and other courses), the score was 3.59 ± 0.24. To see the impact of different educational qualifications on job satisfaction, an ANOVA test was done. The differences were statistically non-significant (p = 0.082) (Table 11).

**Table 11:** Mean MSQ score in different education level regarding job satisfaction (n=217) MSQ: Minnesota Satisfaction Questionnaire

Educational status	Mean ± SD	p-value
MBBS	3.68 ± 0.22	0.082
FCPS	3.66 ± 0.24	
MS	3.53 ± 0.23	
MD	3.68 ± 0.17	
Others	3.59 ± 0.24	

The mean MSQ score regarding job satisfaction in different work experience groups was found to be almost similar. The respondents with the highest mean ± SD 3.68 ± 0.14 have working experience of 16 to 20 years. The second highest mean ± SD 3.67 ± 0.23 was for the group with 11 to 15 years of work experience. The group with work experience up to five years has a mean ± SD 3.63 ± 0.23. Next, the respondents with a mean ± SD 3.59 ±0.24 were those with a working experience of six to 10 years. The respondents with the least mean ± SD 3.47 ± 0.20 have work experience below 20 years. To detect the statistical significance, an ANOVA test was done. The differences were statistically non-significant (p=0.322) (Table 12).

**Table 12:** Mean MSQ score regarding job satisfaction in different work experience groups (n=217) MSQ: Minnesota Satisfaction Questionnaire

Experience	Mean ± SD	p-value
Up to 5 years	3.63 ± 0.23	0.322
6-10 years	3.59 ± 0.24	
11-15 years	3.67 ± 0.23	
16-20 years	3.68 ± 0.14	
>20 years	3.47 ± 0.20	

## Discussion

This cross-sectional descriptive study focused on evaluating the satisfaction of doctors regarding the working environment in selected tertiary hospitals. The study showed that half of the respondents (50%) were in the age group of 31 to 35 years. Another study mentioned that among the healthcare professionals of combined military hospitals in Bangladesh, more than two-fifths (41.1%) were in the age group of 26 to 35 years [9]. About three-fifths of the respondents (58.1%) were male. Most of the respondents (85.3%) were Muslims, and this is a reflection of the religious culture of Bangladesh. 

The study also showed that a maximum of 84.3% of respondents were married. Age, gender, and marital status have often been studied as the underlying variables of job satisfaction. According to this study, no significant association was found between job satisfaction and socio-demographic profile (p>0.05). Earlier research has found a significant relationship between job satisfaction and age, gender, and marital status, while others have discarded any such relationship [10]. In this study, 47.5% of respondents had work experience ranging from six to 10 years. The responders with the highest mean (± SD), 3.68 (±0.14), had a working experience of 16 to 20 years, though no significant association was found between working experience and job satisfaction (p>0.05). A study by Altuntaş reported lower job satisfaction in research assistants, assistant professors, and instructors with less than 10 years of work experience and instructors working on their PhD theses or doing contract work [11]. According to our study findings, two-fifths (41.9%) of the respondents worked for eight hours, more than one-third (34.1%) worked for 10 hours, and about a quarter (24%) of the doctors worked for 12 hours. In another study by Suozzo, the author argued that irrespective of the number of working years and work experience, working in excess of 60 hours per week (even once) as a faculty member reduces the mean job satisfaction [12]. Nikic et al. conducted a survey at the Clinical Center Nis in Serbia, which showed that most health workers found their job stimulating and interesting, but that they worked very hard [13]. Increased workload may result in a higher level of dissatisfaction. When questioned about their satisfaction with the extra workload brought on by the COVID-19 crisis, two-fifths (40.1%) of respondents said they were dissatisfied, and more than one-tenth (12.9%) of the respondents said they were highly dissatisfied. However, no significant association was found between working hours and job satisfaction (p>0.05).

A satisfaction score was also calculated for each of the 20 items on the MSQ scale. The highest mean (± SD) was 4.68 (± 0.62) which was regarding activity, and the lowest mean (± SD) was 2.19 (± 1) was about working conditions. The general mean (± SD) was 3.62 (± 0.23). Another study showed the average MSQ score of all surveyed doctors was 3.11±0.87. The three highest-scored items for doctors were the way company policies were put into practice, the working conditions, and praise for doing a good job [8]. Comparing the mean MSQ score in three different hospitals (DMC, SSMC, ShMC), the highest mean± SD of 3.65± 0.21 was found in SSMC. The DMC scored the second-highest mean of 3.61 ± 0.26, and the ShMC scored the lowest mean of 3.60 ± 0.23.

About two-thirds of the respondents (63.1%) reported an average level of satisfaction regarding general working conditions. Out of 217 respondents in this study, less than one-third (30.4%) had high satisfaction, and more than two-thirds (69.6%) had average satisfaction regarding the physical working environment. The study showed that with regard to the psychosocial environment, the majority (93.1%) of doctors reported high levels of satisfaction, while 6.9% reported average levels of satisfaction. Another study conducted during the COVID-19 pandemic showed similar findings. [14]

Evidence shows that multiple workplace-related factors affect the effectiveness of people who work there. These can include the working space, water supply, electricity supply, ventilation system, cleanliness facility, infection control facility, availability of PPE, restroom condition, etc. An organization’s overall productivity and quality of individual work influence job satisfaction [15]. This study found that satisfaction regarding cleanliness, infection control, and working space was 58.6%, 49.3%, and 37.3%, respectively. When asked about the facilities available at the workplace, nearly three-fifths of the doctors (59.4%) felt that their workplace was poorly equipped and had scope for improvement. In this study, three-quarters (75.1%) of the doctors were deprived of getting PPE from the hospital. To protect themselves and their patients from the transmission of germs and infectious diseases, PPE is essential in any pandemic event.

Job design seeks to integrate means by which job characteristics can be changed, like workload, work variety, and workplace supervisory support, which will lead to enhanced worker satisfaction and hence increased performance [16]. In our study, two-thirds of the participants (66.9%) were not satisfied with their salary in relation to their workload. Doctors were expecting a handsome salary during the COVID situation due to the huge workload. In our study, according to information about satisfaction with the workload, more than half the respondents (53%) were dissatisfied. Another study showed that the positive working environment, low fringe benefits, and poor salaries were the main factors behind the job satisfaction of health workers working in health organizations in the public sector, while socio-demographic characteristics were found to have no significant relationship with their job satisfaction [17]. According to Abdullah et al., salary and benefits were key factors in employee satisfaction and turnover. [18]

In our study, satisfaction regarding job retention, the right position, promotion, and health insurance was 81.6%, 63.6%, 46.5%, and 10.1%, respectively. Another study showed that the right position and promotion opportunities, benefits, health insurance, and rewards reflect job satisfaction among public healthcare workers in Pakistan [19]. Doctors were frontline fighters during COVID-19, and their participation in the fight against this pandemic is unusual. During a pandemic, doctors assumed a considerable risk in providing patient care, yet they were not provided with health insurance.

The information gathered was challenging to obtain since some of the participants were hesitant to share their genuine opinions. This study may be a source for future studies in different hospitals with a vast scope.

## Conclusions

The COVID-19 pandemic was a major public health threat. The role of doctors was crucial, and they served as frontline soldiers. Unsatisfactory working conditions restrict doctors from rendering their capabilities. To fulfill the guidelines of care during COVID-19 or any pandemic situation, doctors need a workplace that permits them to work enthusiastically. This study revealed that about two-thirds of the respondents in the current study reported an average level of satisfaction. Satisfaction regarding the psychological environment was higher among the respondents than that regarding physical working conditions. To ensure that doctors are as satisfied as possible during pandemics and to provide quality care to patients, hospitals must use evidence-based interventions.
